# Caveolin1 Identifies a Specific Subpopulation of Cerebral Cortex Callosal Projection Neurons (CPN) Including Dual Projecting Cortical Callosal/Frontal Projection Neurons (CPN/FPN)

**DOI:** 10.1523/ENEURO.0234-17.2017

**Published:** 2018-01-18

**Authors:** Jessica L. MacDonald, Ryann M. Fame, Eva M. Gillis-Buck, Jeffrey D. Macklis

**Affiliations:** 1 Department of Stem Cell and Regenerative Biology, Center for Brain Science, and Harvard Stem Cell Institute, Harvard University, Cambridge, MA 02138; 2 Department of Biology, Syracuse University, Syracuse, NY 13244

**Keywords:** axonal projections, connectivity, corpus callosum, neocortex, neuronal subtypes

## Abstract

The neocortex is composed of many distinct subtypes of neurons that must form precise subtype-specific connections to enable the cortex to perform complex functions. Callosal projection neurons (CPN) are the broad population of commissural neurons that connect the cerebral hemispheres via the corpus callosum (CC). Currently, how the remarkable diversity of CPN subtypes and connectivity is specified, and how they differentiate to form highly precise and specific circuits, are largely unknown. We identify in mouse that the lipid-bound scaffolding domain protein Caveolin 1 (CAV1) is specifically expressed by a unique subpopulation of Layer V CPN that maintain dual ipsilateral frontal projections to premotor cortex. CAV1 is expressed by over 80% of these dual projecting callosal/frontal projection neurons (CPN/FPN), with expression peaking early postnatally as axonal and dendritic targets are being reached and refined. CAV1 is localized to the soma and dendrites of CPN/FPN, a unique population of neurons that shares information both between hemispheres and with premotor cortex, suggesting function during postmitotic development and refinement of these neurons, rather than in their specification. Consistent with this, we find that *Cav1* function is not necessary for the early specification of CPN/FPN, or for projecting to their dual axonal targets. CPN subtype-specific expression of *Cav1* identifies and characterizes a first molecular component that distinguishes this functionally unique projection neuron population, a population that expands in primates, and is prototypical of additional dual and higher-order projection neuron subtypes.

## Significance Statement

Callosal projections neurons (CPN) are a diverse set of neocortical interhemispheric excitatory projection neurons that integrate multiple distinct brain regions. While retrograde studies have identified CPN subpopulations that project to multiple targets, molecular identifiers are lacking for these subpopulations. Here, we identify Caveolin 1 (*Cav1*) as an identifier of CPN with dual callosal and ipsilateral frontal projections (CPN/FPN). CAV1 is expressed by over 80% of these CPN/FPN, with expression peaking early postnatally. CAV1 localizes to the soma and dendrites of CPN/FPN, suggesting function during postmitotic development and refinement of these neurons, rather than in their early specification. Identification of this molecular identifier of CPN/FPN will enable future functional investigations of this unique neuronal population.

## Introduction

The neocortex is composed of many distinct subtypes of neurons that developmentally form precise subtype-specific connectivity and circuitry to enable cortex to perform its many complex functions of sensory processing, associative integration, cognition, and motor output. Callosal projection neurons (CPN) are the broad population of commissural neurons that connect the cerebral hemispheres via the corpus callosum (CC). CPN are excitatory pyramidal projection neurons whose cell bodies reside in neocortical Layers II/III (∼80% in mouse), V (∼20%), and a few % in VI (Ivy and Killackey, 1981; Silver et al., 1982; Catapano et al., 2001; Fame et al., 2011; Greig et al., 2013; MacDonald et al., 2013) and play multiple roles in complex associative and integrative cognition. CPN are a diverse set of subpopulations, distinguished by characteristics including cell body location, birthdate, electrophysiological and neurochemical properties, dendritic tree distribution, axonal target(s), and molecular expression (Aboitiz and Montiel, 2003; Mitchell and Macklis, 2005; Benavides-Piccione et al., 2006; Molyneaux et al., 2009; Fame et al., 2011; Otsuka and Kawaguchi, 2011; Fame et al., 2016a). While these properties describe and categorize CPN, they also determine the function(s) of diverse CPN subtypes.

Molecular controls that both define and regulate development of these distinct CPN subtypes are just beginning to be elucidated. A combinatorially-expressed set of genes that both define CPN as a broad population and distinguish novel subpopulations of CPN during development has been identified (Molyneaux et al., 2009), and a few molecular controls have been investigated functionally. For example, SATB2 regulates CPN identity throughout the neocortex; in the absence of its function, CPN project axons subcortically rather than across the CC, and they take on some molecular characteristics of corticofugal projection neurons (Alcamo et al., 2008; Britanova et al., 2008). Other molecular controls regulate specific aspects of CPN development, and/or distinct subpopulations. *Cux1* and *Cux2* regulate dendritic complexity and synapse formation of Layer II/III CPN (Cubelos et al., 2010), and *Cux1* regulates activity-dependent interhemispheric connectivity of CPN (Rodríguez-Tornos et al., 2016). CITED2 regulates generation of Layer II/III CPN throughout the neocortex, as well as areal identity and neuronal connectivity specifically of somatosensory CPN (Fame et al., 2016b). CTIP1, on the other hand, regulates specification of deep layer CPN (Woodworth et al., 2016).

Caveolin 1 (*Cav1*) was identified in Molyneaux, et al. (2009) as a CPN subpopulation-restricted gene during neocortical development, with a peak of expression at postnatal day 3 (P3). *Cav1* encodes a membrane-bound scaffolding protein necessary for a range of cellular processes, including functions related to the unique lipid raft structures it forms, caveolae. *Cav1* is highly expressed broadly in developing blood vessels and endothelial cells, and is required for their proper development (Razani et al., 2001; Schubert et al., 2002), and for blood-brain barrier permeability (Zhao et al., 2014). While caveolae are not thought to exist in neurons (Head and Insel, 2007), *Cav1* knock-out mice exhibit neurologic abnormalities including limb clasping, abnormal spinning, muscle weakness, reduced activity, and gait abnormalities (Trushina et al., 2006).

As a lipid raft scaffolding molecule, CAV1 interacts with multiple binding partners, some of which are particularly compelling as components of neuronal function. CAV1 binds directly to the calmodulin-dependent scaffolding protein striatin (Gaillard et al., 2001), which acts as a signaling platform in dendritic spine signal transduction (Benoist et al., 2006), and SNAP25, which complexes to CAV1 presynaptically on synaptic potentiation (Braun and Madison, 2000). CAV1 plays roles in neurotrophin response, and has the ability to interact with synaptosome complexes (Bilderback et al., 1997, 1999; Head and Insel, 2007). CAV1 is required for estrogen receptor α (ERα) activation of the metabotrophic glutamate receptor, mGluR1α, in hippocampal neurons, potentially acting in long-term depression (Takayasu et al., 2010). CAV1 provides a scaffold for both the ERα voltage-dependent anion receptor and one of its interactors, the IGF-1 receptor (Maggi et al., 2002; Marin et al., 2009), a known positive regulatory pathway for corticospinal projection neuron axonal outgrowth (Ozdinler and Macklis, 2006).

*Cav1* has additionally been implicated in neuronal differentiation from progenitors (Li et al., 2011), synaptic ribbons in photoreceptors (Kachi et al., 2001), and other neurotransmitter receptor functions including muscarinic cholinergic receptors and NMDA receptor NR2B (Lai et al., 2004). Further, CAV1 interacts with Rho-family GTPase RAC1, and this interaction has been directly implicated in neurite outgrowth (Kang et al., 2006). These neuronal-specific processes might account for some of the neurologic deficits in *Cav1* loss-of-function mice described above, and additionally motivate this study of *Cav1* in CPN.

Here, we further identify that *Cav1* is highly expressed during axonal and dendritic development in a laminarly- and regionally-restricted subset of dual projecting CPN that extend both homotopic axonal connections to mirror image locations in the contralateral hemisphere, and rostral connections to ipsilateral frontal areas, sending information from sensory or motor functional areas to higher hierarchical cortical areas (here, these dual-projecting neurons are referred to as CPN/frontal projection neurons (FPN; Mitchell and Macklis, 2005). The majority of these CPN/FPN are located in neocortical Layer Va in both the primary somatosensory area (S1) and in a large expansion in caudo-lateral secondary somatosensory neocortex (S2; Mitchell and Macklis, 2005). While CPN/FPN have been identified anatomically, no molecular controls over this population’s unique connectivity or function have been identified, limiting understanding of the development and function of these specialized dual-projecting neurons. Our investigations of *Cav1* expression and function reported here identify and characterize a first molecular component that distinguishes and might control aspects of this functionally unique projection neuron population.

## Materials and Methods

### Mouse lines

All animal procedures were performed in accordance with the Harvard University animal care committee’s regulations. *Cav1-*null mice on a congenic C57/Bl6 background (B6.Cg-*Cav1^tm1Mls^*/J) were obtained from The Jackson Laboratory, strain number 007083 (RRID:IMSR_JAX:007083). The original targeted null mutation was generated by Michael Lisanti at The Albert Einstein College of Medicine. A 2.2-kb region of the gene including exons 1 and 2 and a portion of the promoter region was replaced with a neomycin resistance cassette via homologous recombination (Razani et al., 2001).

BTBR acallosal mice on a congenic background (BTBR *T* ^+^ *tf*/J) were obtained from The Jackson Laboratory, strain number 002282 (RRID:IMSR_JAX:002282; Wahlsten et al., 2003). LP/J mice have been shown to be an appropriate callosal control population for BTBR mice. LP/J mice (LP/J) were obtained from The Jackson Laboratory, strain number 000676 (RRID:IMSR_JAX:000676).

C57/Bl6 wild-type (WT) mice were obtained from The Jackson Laboratory (RRID:IMSR_JAX:000664), and were used to breed with *Cav1***-**null mice, and for birthdating and electroporation experiments. FEZF2 mutants were generated by Hirata et al. (2004; GenBank accession number: AB042399).

### Immunocytochemistry

Immunocytochemistry was performed as follows. Briefly, brains were fixed by transcardial perfusion with PBS, followed by 4% paraformaldehyde, and postfixed overnight at 4°C in 4% paraformaldehyde. Brains were sectioned at 50μm on a vibrating microtome (Leica). Sections were blocked in 0.3% BSA (Sigma), 8% goat or donkey serum, and 0.3% Triton X-100 (Sigma) for 1 h at room temperature (RT), before incubation in primary antibody. Secondary antibodies were selected from the Alexa Fluor series.

Antigen retrieval methods were required to expose antigens for some of the primary antibodies, including CAV1. Sections were incubated in 0.1 M citric acid (pH 6.0) for 10 min at 95-98°C, and sections were rinsed in PBS before blocking. For thymidine analogues (IdU, CldU), HCl antigen retrieval was required. Tissue was rinsed quickly in ddH_2_O and incubated in 2 N HCl for 2 h at RT, and sections were rinsed in PBS before blocking.

Primary antibodies and dilutions were used as follows: rabbit anti-CAV1, 1:500 (Cell Signaling #3238; RRID:AB_2072166); goat anti-LMO4, 1:200 (Santa Cruz Biotechnology SC- 11122; RRID:AB_648429); rat anti-CTIP2 1:500 (Abcam ab18465; RRID:AB_2064130), mouse anti-BrdU, 1:500 (Becton Dickinson #347580; clone B44; RRID:AB_10015219; detects IdU); rat anti-BrdU, 1:500 (Accurate #OBT- 0030; clone BU1/75; RRID:AB_2313756; detects CldU); rabbit anti-GFP, 1:500 (Invitrogen; RRID:AB_221569); mouse anti-SATB2, 1:500 (Abcam ab51502; RRID:AB_882455); goat anti-BHLHB5, 1:200 (Santa Cruz Biotechnology SC-6045; RRID:AB_2065343); and rabbit anti-5-HT, 1:1000 (Immunostar 20080; RRID:AB_572263).

### In situ hybridization

Nonradioactive colorimetric *in situ* hybridization was performed using probes labeled with digoxigenin (dig)-UTP generated by reverse transcription PCR. The probe sequence for *Cav1* was previously published (Molyneaux et al., 2009). Postnatal tissue was fixed overnight in 4% paraformaldehyde at 4°C. Fixed tissue was sectioned on a VT1000S vibrating microtome (Leica Microsystems) to a thickness of 50 μm. Embryonic tissue was flash frozen in 2-methyl butane, embedded in TBS, and cryosectioned on a CM3050S cryostat (Leica Microsystems) to a thickness of 14 μm. Sense probes were used as negative controls in all experiments. Sections were mounted on Superfrost plus slides (Fisher Scientific) and postfixed in 4% PFA in PBS for 10 min, rinsed in PBS for 3 min, permeabilized in 0.3% Triton X-100 (Sigma) followed by RIPA cell lysis buffer (150 mM sodium chloride, 1% Triton X-100, 1% deoxicholic acid sodium salt, 0.1% sodium dodesil sulfate, 50 mM Tris-HCl, pH 7.5, and 2 mM EDTA), refixed in 4% PFA, acetylated for 15 min in 0.1 M triethanolamine/0.4% HCl/0.25% acetic anhydride (Sigma), and then preybridizied in 65°C hybridization buffer [50% formamide, 5× SSC, 5× Denhardts (1 μg/ml Ficoll 400, 1 μg/ml polyvinylpyrrolidone, 1 μg/ml BSA), 500 μg/ml sheared salmon sperm DNA, and 250 μg/ml yeast RNA]. Slides were incubated overnight (14-20 h) at 65°C in 2 μg/17 ml dig-labeled probe in hybridization buffer in a plastic mailer. Slides were then subjected to stringency washes in 2× SSC/50% formamide/0.1% Tween 20 at 65°C for 2 h. Sections were then rinsed in MABT [0.9 M maleic acid (Sigma), 0.1 M NaCl (Sigma), 0.0005% Tween 20 (Sigma), and 0.175 M NaOH (Sigma)] at RT, blocked in 10% goat serum in MABT, and incubated overnight in goat alkaline phosphatase-conjugated anti-dig (1:1000; Roche) primary antibody in block. The following day, the slides were rinsed with MABT, followed by a 30-min wash in alkaline phosphatase reaction buffer (100 mM Tris, pH 9.5, 50 mM MgCl_2_, 100 mM NaCl, and 0.1% Tween 20). The alkaline phosphatase reaction was developed with 0.25 mg/ml nitro-blue tetrazolium (NBT)/125 μg/ml 5-bromo-4-chloro-3'-indolyphosphate (BCIP) in phosphatase reaction buffer, changing to fresh solution every 1-4 h at RT or every 6-9 h at 4°C. When the reaction was complete, tissue was rinsed in 0.1% Tween 20 in PBS, postfixed in 4% PFA for 30 min.

### Retrograde labeling of cortical projection neurons

#### Perinatal retrograde labeling of CPN, corticospinal motor neuron (CSMN), Corticostriatal projection neurons (CStrPN), and Anterior commissure projecting neurons (ACN)

Perinatal retrograde labeling of CPN, and CSMN was performed using a Vevo 770 ultrasound backscatter microscopy system (VisualSonics). Briefly, P1 pups of either sex were anesthetized by hypothermia, and CC, or pons, respectively, were injected under ultrasound guidance using a pulled glass micropipette (tip diameter, 80–100 μm), and cell bodies were labeled with the β-subunit of cholera toxin (CTB) labeled with Alexa Fluor dye (2 mg/ml, Invitrogen). For CSMN, six 23-nl injections of cholera toxin subunit B (2 µg/µl) were deposited bilaterally into the pons. The brains were harvested 2 d after injection (P3).

Because CStrPN reach the striatum later than P1, pups were anesthetized with hypothermia and injected sterotaxically at P3. The striatal injection point is very close to the anterior commissure in the developing brain, so both CStrPN and ACN were labeled simultaneously. The injections were made at an angle of 32.5° from horizontal, 3 mm posterior to bregma, and 1.8 mm lateral (left). The CTB labeled with Alexa Fluor dye (2 mg/ml, Invitrogen) was injected at a depth of 4.2 mm, and 4.6 nl of dye was delivered three times (total of 13.8 nl) into three injection sites at depths, 4.2, 4.1, and 4.0 mm. The needle was removed after 5 min to allow diffusion and avoid retracting the dye with the needle. The brains were harvested 2 d after injection (P5).

#### Postnatal dual CPN/FPN and CPN/BPN retrograde labeling

P6 pups were anesthetized with hypothermia. P21 mice were deeply anesthetized with Avertin (0.02 ml/g body weight, i.p.). Tracers were injected transcranially with sharp pulled glass micropipettes (tip diameter 80-100 μm) in presumptive premotor and sensory-motor areas, as described below. Double fluorescent tracer injections were performed to label simultaneously: (1) CPN in sensory-motor cortex, and (2) FPN with long-distance ipsilateral projections to the premotor cortex. CPN with projections to the contralateral neocortex were labeled with Alexa Fluor 647-conjugated cholera toxin subunit-β (2 mg/ml, Invitrogen) with 25 injection sites, 46 nl (10 injections of 4.6 nl) each site at a depth of 250 μm at P6 and 450 μm at P21. The most caudal injection site was 1 mm caudal to bregma, and the most rostral injection site was ∼1 mm caudal to the olfactory bulbs. All other injections were evenly spaced to complete the 5 × 5 grid. Ipsilateral corticocortial projections to the premotor cortex were simultaneously retrogradely labeled with injections of Alexa Fluor 555-conjugated cholera toxin subunit B (2 mg/ml, Invitrogen) with seven injection sites, 46 nl each site and a depth of 250 μm at P6 and 450 μm at P21. Brains were harvested 2 d after injection (P8).

Backward projecting neurons (BPNs) were retrogradely labeled via transcranial injection of cholera toxin subunit B (2 mg/ml, Invitrogen) at P7 into the ipsilateral caudal cortex, covering the area of the presumptive somatosensory cortex. The most rostrolateral injection site was 1.5 mm rostral to lambda, and 2.5 mm lateral to the midline. All other injections were evenly spaced to complete the 3 × 4 grid. Each mouse received ten 4.6 nl volume injections at 12 injection sites. For each injection site, five injections were made at 200 μm depth from the dorsal surface of the brain, two injections at 150 μm depth, 1 injection at 100 μm depth, and two injections at 50 μm depth. Brains were harvested 2 d after injection (P9).

Small punctures were made in the skull at the location of each injection point with either a pulled glass pipette (P6) or a fine suture needle (P21) before lowering the injection needle to the proper depth. This avoided the need for large craniotomies, thus minimizing insult and enhancing recovery time, while allowing for exact depth measurements to be made accurately. For all retrograde labeling experiments, our approaches minimized surgical time and insult; thus, morbidity was very low, and the survival rate was near 100%.

### Birthdating

For IdU and CldU birthdating, equimolar delivery of IdU (57.5 mg/kg) or CldU (42.5 mg/kg) was performed by intraperitoneal injections at 12-h intervals from embryonic day (E) E11 to E15.5 (Vega and Peterson, 2005), calculating embryonic age with E0.5 as the morning of observed vaginal plug. Mice of either sex were perfused at P6, and brains prepared for immunocytochemistry.

### Gain-of-function constructs

For control gain-of-function experiments, a vector containing a constitutively active CMV enhancer/β actin promoter driving GFP downstream of an internal ribosomal entry site (IRES) was used (*GFP^control^*, generous gift of C. Lois, MIT (Molyneaux et al., 2005). For the *Cav1* overexpression construct, called *Cav1^GFP^*, full-length *Cav1* cDNA was cloned into the same vector backbone using a Sal1/Not1 digest (New England Biolabs, Ipswich, MA) of the *Cav1* cDNA from a pSport1 vector purchased from Open Biosystems (clone ID 30062454). A sequenced clone with perfect alignment to the NCBI reference sequence NM_007616 in both the sense and antisense orientations was selected for experiments.

### In utero electroporation

*In utero* electroporations were performed essentially as described previously (Saito and Nakatsuji, 2001; Molyneaux et al., 2005; Saito, 2006). Timed pregnant mice were deeply anesthetized with isofluorane. Hair was removed from the abdomen, and the dam was secured supine on a heated surgical platform. Individual pups were withdrawn from the abdominal cavity and moistened with sterile 37°C 1× PBS. Using a Vevo 770 ultrasound backscatter microscopy system (VisualSonics) to visualize the lateral ventricles, a beveled glass micropipette (50 μm width) was inserted into one lateral ventricle of each injected pup, and fifteen 69 nl volume injections of prepared DNA mixture were delivered to the ventricle. Soon after retracting the glass micropipette, electric current was applied to the head of the embryo through two 1-cm diameter platinum electrodes, orienting the current to drive the negatively charged DNA into the dorsal telencephalon. Five 25-V pulses of 50 ms duration at 1-s intervals were delivered using a CUY21EDIT square wave electroporator (Nepa Gene). Each injected embryo was gently returned to the abdominal cavity, and the next selected embryo was carefully withdrawn. No more than six embryos of either sex were injected per dam. The abdomen was sutured. Dams recovered on a warm heating pad, and once ambulatory were administered 250 μl of buprenorphine (0.015 mg/ml sterile PBS) before returning to their cages.

### Quantification of FPN/CPN

For P8 quantification of CAV1-expressing CPN/FPN, anatomically matched sections were selected (*n* = 4 WT), and CAV1 immunocytochemistry was performed. Digital boxes of fixed width indicated each of the four cortical regions, and the number of CPN/FPN and CAV1^+^ CPN/FPN were counted. Percentages of FPN/CPN that express CAV1 in each region were calculated from total numbers. Error bars or ± indicate the SEM. Individuated neuronal somata were counted for each assignment; it was not possible to count all CPN or CAV1-expressing neurons in some densely packed microdomains due to high neuronal density.

For P8 quantification of CPN/FPN in *Cav1*-null mice, anatomically matched sections were selected (*n* = 6 WT, *n* = 6 *Cav1* ^–/–^). Digital boxes of fixed width indicated the S1 or S2 cortical regions, and the number of CPN/FPN, and FPN were counted in S1 and S2. The percentage of FPN with concurrent callosal projections was also calculated. No significant differences were detected between WT and *Cav1* ^–/–^. Error bars or ± indicate the SEM. Data were analyzed by unpaired Student’s *t* test. Statistical analyses were performed in Prism (GraphPad Software, Inc.). A two-tailed *post hoc* observed power was calculated from Cohen’s *d*, the *p* value, and *n* (Table 1).

**Table 1.** Statistical Analyses Performed

	Data structure	Type of test	*n* (WT; KO)	*p* value	Observed power	95% confidence interval
Fig. 6G, S1	Gaussian	Unpaired *t* test	(6; 6)	0.3585	0.4391	−9.642–24.31
Fig. 6G, S2	Gaussian	Unpaired *t* test	(6; 6)	0.2831	0.4278	−21.05–64.72
Fig. 6H, S1	Gaussian	Unpaired *t* test	(6; 6)	0.4056	0.4316	−111.2–253.2
Fig. 6H, S2	Gaussian	Unpaired *t* test	(6; 6)	0.3004	0.4050	−18.72–54.72
Fig. 6I, S1	Gaussian	Unpaired *t* test	(6; 6)	0.4529	0.4529	−0.3069–0.1476
Fig. 6I, S2	Gaussian	Unpaired *t* test	(6; 6)	0.6214	0.6214	−0.1722–0.1081

### Microscopy and image analysis

Whole mount images were acquired using an SMZ1000 fluorescence dissecting microscope (Nikon) with a SPOT CCD digital camera (Diagnostic Instruments) and SPOT acquisition software.

Tissue sections were imaged on a Nikon E1000 microscope (Nikon Instruments) equipped with an XCite 120 illuminator (EXFO) and Q-imaging Retixa EX cooled CCD camera (Q-imaging Corp), or a Nikon 90i microscope using a 1.5-megapixel cooled CCD digital camera (Andor Technology), a five-megapixel color CCD digital camera (Nikon Instruments). Images were collected and analyzed with Volocity image analysis software (version 4.0.1; Improvision Inc.) or Elements acquisition software (Nikon Instruments).

Laser confocal analysis was performed using a Bio-Rad Radiance 2100 confocal microscope with LaserSharp2000 imaging software (Bio-Rad Laboratories). Images were processed using a combination of functions provided by ImageJ (Rasband, W.S., ImageJ; http://imagej.nih.gov/ij/, 1997-2011.) and Adobe Photoshop/Illustrator software packages (Adobe).

## Results

### CAV1 is expressed by a restricted population of CPN, and is excluded from CSMN

**Figure 1.**
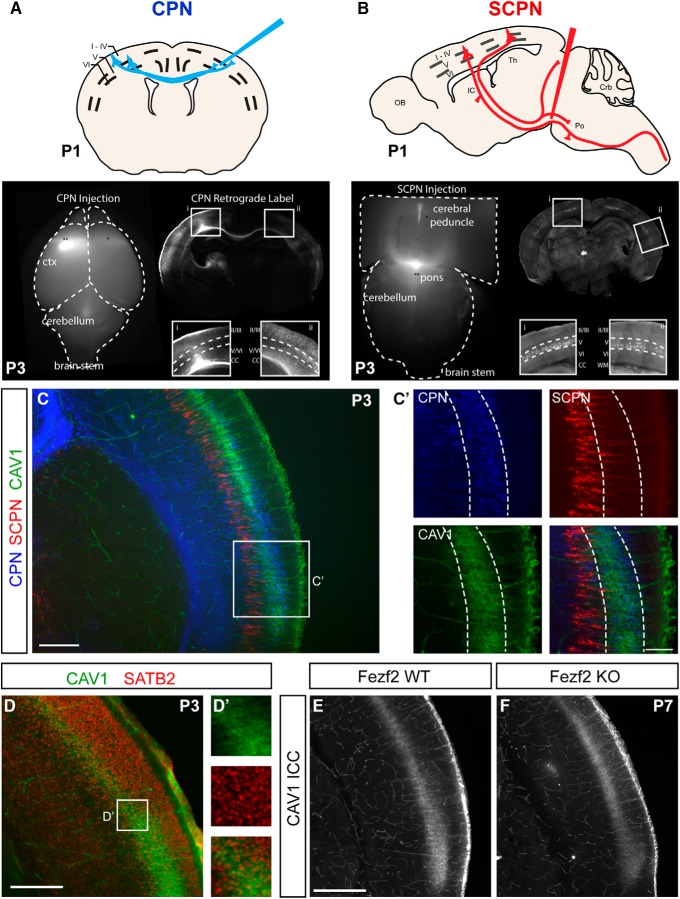
CAV1 is expressed by CPN and excluded from SCPN at P3. ***A***, ***B***, Retrograde labeling was performed at P1 by injecting CTB_647_ into the contralateral hemisphere to label CPN (***A***) and CTB_555_ into the pons to label SCPN (***B***). Example images of retrogradely-labeled brains are shown at P3, including wholemount images of the injection sites (dorsal view for CPN, ventral view for SCPN) and coronal sections showing the laminar distribution of the labeled neurons. ***C***, ***C’***, CAV1 (green) immunocytochemistry at P3 reveals that CAV1 is expressed in Layer V, with a caudo-lateral distribution similar to SCPN (red). However, CAV1 (green) colocalizes with retrogradely labeled CPN (blue) in Layer Va, and it is excluded from SCPN (red) in Layer Vb. The box in ***C*** indicates the region of higher magnification images in ***C’***. ***D***, CAV1 expression (green) overlaps with the CPN developmental control SATB2 (red), reinforcing that CAV1 is expressed by CPN. ***E***, ***F***, CAV1 expression is not altered in *Fezf2*-null cortex (***F***), which is developmentally devoid of SCPN, reinforcing that CAV1 is not expressed by SCPN. Scale bars: 250 μm (***C***), 100 μm (***C’***), 250 μm (***D***), and 500 μm (***E***, ***F***); IC, internal capsule; Th, thalamus; OB, olfactory bulb; Po, pons; Crb, cerebellum; roman numerals indicate cortical layer.

**Figure 2.**
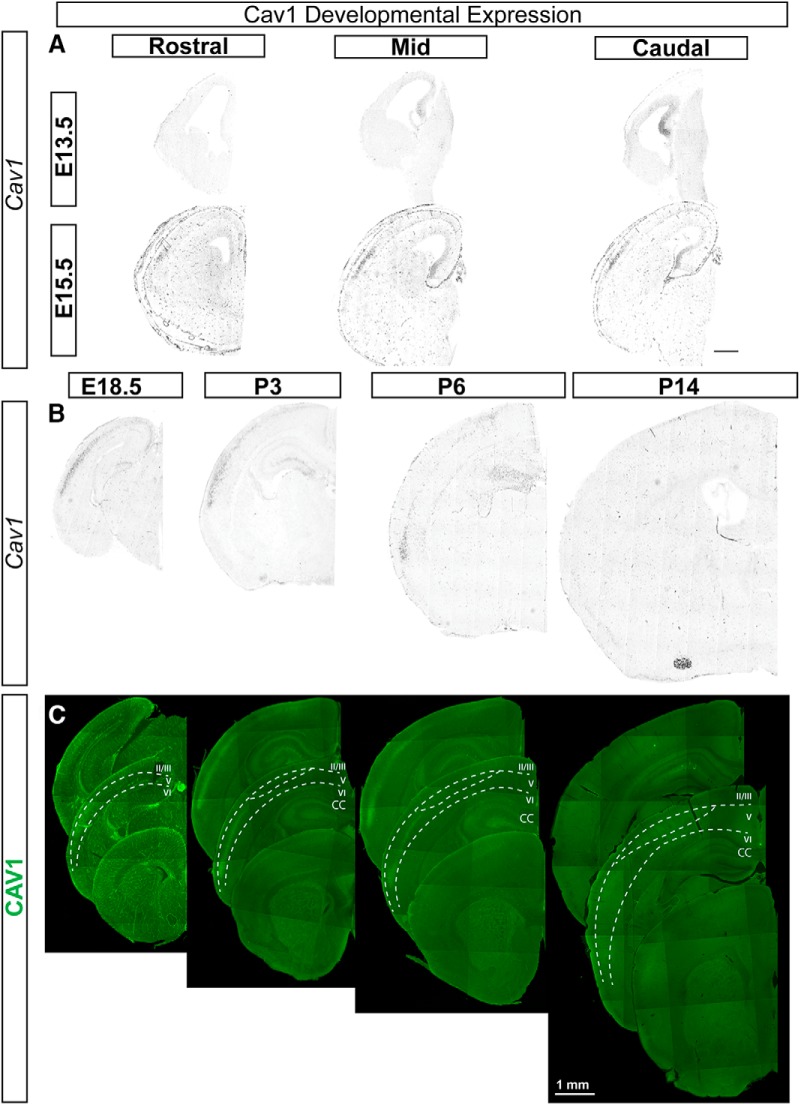
CAV1 is expressed in caudo-lateral cortex during mid-stage cortical development. ***A***, *Cav1* mRNA is not detected in pallial progenitors or cortical plate at E13.5 but is detected in the caudal cortical hem. By E15.5, *Cav1* is expressed in the caudo-lateral cortical plate. ***B***, To determine whether *Cav1* expression follows a developmental gradient, we examined expression at later embryonic stages through postnatal development. *Cav1* mRNA is expressed by a restricted population of Layer Va cells (presumptive CPN) in the caudo-lateral cortex from late embryonic stages (E18.5) through early postnatal development (P3, P6), with expression reduced in the cortex by P14. ***C***, CAV1 protein is detected in a similar pattern to *Cav1* mRNA throughout development. CAV1 is expressed by CPN within the same caudo-lateral Layer Va region throughout late embryonic and early postnatal development, suggesting that it is not following a developmental gradient. Scale bar: 1 mm; E, embryonic day.

*Cav1* is highly expressed by CPN relative to CSMN in the developing neocortex, with a peak of expression between P3 and P6 (Molyneaux et al., 2009), during the critical time when CPN are making axonal and dendritic connections. *Cav1* is not detected in CPN by P14. We more thoroughly investigated CAV1 expression by immunocytochemistry, and similarly identified that CAV1 is indeed expressed by CPN in cortical Layer Va, and is excluded from subcerebral projection neurons (SCPN; of which CSMN are a subpopulation) by combining CAV1 immunocytochemistry and dual retrograde labeling of CPN (from the contralateral cortex) and SCPN (from the pons; Fig. 1*A–C*). The CAV1-expressing population of CPN is clearly superficial to the SCPN (Fig. 1*C*), and CAV1 is coexpressed with SATB2 (Fig. 1*D*), which is expressed by CPN and excluded from SCPN. CAV1 expression is unchanged in *Fezf2* loss-of-function neocortex, in which no subcerebral neurons are specified (Molyneaux et al., 2005), confirming that CAV1 is excluded from CSMN (Fig. 1*E*,*F*). The highly restricted expression pattern suggests that *Cav1* functions in a very specific subpopulation of CPN, rather than playing a broad role in CPN development (Figs. 1, 2).

Developmentally, *Cav1* mRNA is not detected in pallial progenitors or the cortical plate at E13.5, but it is detected in the caudo-lateral cortical plate by E15.5 (Fig. 2*A*). Both *Cav1* mRNA and CAV1 protein are detected in cortical neurons at E18.5 (in addition to developing blood vessels), are highly expressed at P3 and P6, and are no longer detectable by P14 (Fig. 2*B*,*C*). *Cav1*-expressing neurons are distributed uniquely in cortical Layer Va, especially in caudo-lateral areas. This pattern is not a developmental gradient, and is maintained specifically at all developmental ages at which *Cav1* is expressed (Fig. 2).

**Figure 3.**
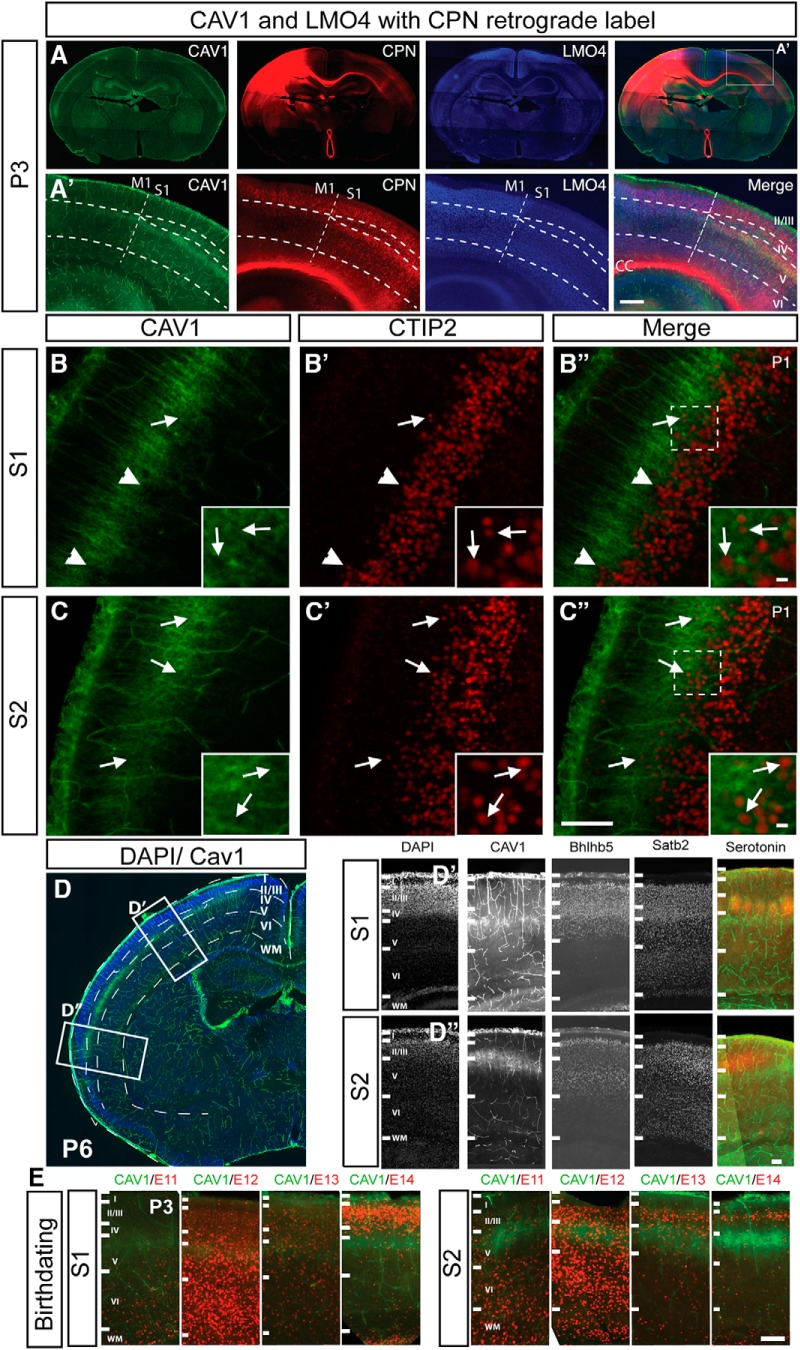
CAV1 is expressed by Layer Va CPN in an areally restricted fashion. ***A***, ***A’***, Expression of CAV1 is areally restricted; CAV1 (green) is highly expressed by CPN (red; CTB_555_ retrograde label) throughout primary and secondary somatosensory cortex (S1 and S2). CAV1 expression is not readily detected in CPN within motor cortex (M1), as delineated by Layer II/III expression of LMO4 (blue). ***A’***, Inset from ***A***. Dashed line indicates M1–S1 boundary and cortical lamina. ***B***, In S1, CAV1 (green) is largely excluded from the CTIP2 (red)-expression domain (arrowheads) indicating SCPN, with only a small subpopulation of CTIP2+ve neurons extending into the CAV1-expression domain (arrow). ***C***, In S2, the boundary between CAV1 (green) and CTIP2 (red) is not as clearly defined, with more CTIP2+ve neurons interspersed with CAV1 (arrows). Regions of higher magnification insets are indicated in ***B”*** and ***C’’***. ***D***, At P6, CAV1 is expressed in Layer V, within the Satb2 and Bhlhb5-expressing domain, and below the serotonin-expressing barrel cortex in Layer IV. Regions of higher magnification insets are indicated in ***D’’*** and ***D’’***. ***E***, CAV1-expressing neurons are born between days E12.5 and E13.5 in both S1 and S2. deoxyuridine analogs (CldU or IdU) were injected at 12-h intervals throughout corticogenesis, and immunocytochemistry for BrdU (red) and CAV1 (green) was performed at P3, revealing that CAV1-expressing neocortical neurons, both in S1(somatosensory Layer V) and S2 (caudolateral expansion), are born between E12.3 and E13.5. Scale bars: 500 μm (***A’***) and 100 μm (***B–E***).

Interestingly, CAV1 expression is restricted at P8 to a subpopulation of CPN extending in Layer Va throughout primary somatosensory cortex (S1), and expanding in the caudo-lateral cortex (S2), and auditory cortex (A1) more caudally, but is mostly excluded from motor cortex (M1). This exclusion is clear by comparison between CAV1 immunocytochemistry and LMO4, which is highly expressed by CPN in motor cortex, but excluded from Layer II/III CPN in somatosensory cortex (Azim et al., 2009; Huang et al., 2009; Fig. 3*A*). This area-specific expression further supports subpopulation-specific function of CAV1 in CPN. Because of the strong expression of CAV1 at all developmental ages in Layer Va, particularly in caudo-lateral areas close to archicortex, where canonical cortical neuron subpopulations are not maintained, we investigated whether CAV1-expressing neurons exclude CTIP2, the canonical postmitotic SCPN control transcription factor highly expressed by specific SCPN Layer V populations (Arlotta et al., 2005; Chen et al., 2008). There is very little overlap between CAV1 and CTIP2 in S1 (Fig. 3*B*), although a small subset of CAV1-expressing neurons in far caudo-lateral S2 express CTIP2 (Fig. 3*C*). Based on size and morphology, these CAV1^+^/CTIP2^+^ neurons appear to be pyramidal projection neurons; however, they do not appear to project subcerebrally, as determined by the lack of overlap between CAV1 and retrogradely-labeled SCPN (Fig. 1). This quite unique population of non-SCPN, CTIP2^+^ neurons has not been extensively defined, but they might be a subpopulation of neurons with a transient developmental spinal projection that is later lost (Polleux et al., 2001; Arlotta et al., 2005).

To investigate more deeply what subpopulation(s) of CPN express CAV1, we examined expression of CAV1 in comparison to lamina-restricted molecular markers (Fig. 3*D*). CAV1 is coexpressed with Bhlhb5, which is expressed in Layers II–V, while CAV1 is expressed below the Layer IV barrel field, as indicated by 5-HT expression. Thus, CAV1 is expressed in Layer V. Further, to better delineate the population(s) and to enable optimal targeting of the population via *in utero* electroporation in future experiments, we performed birthdating analysis with thymidine analogs every 12 h throughout corticogenesis. These experiments reveal that CAV1-expressing neurons are born between E12.5 and E13.5, consistent with the dominant birthdate ranges for neocortical neurons residing in Layer V (Fig. 3*E*). CAV1-expressing neurons in caudo-lateral S2 cortex might be born a few hours earlier (peak at E12.5) than those of S1 Layer V (peak at E13.5).

### CAV1 is expressed by over 80% of dual-projecting FPN/CPN

**Figure 4.**
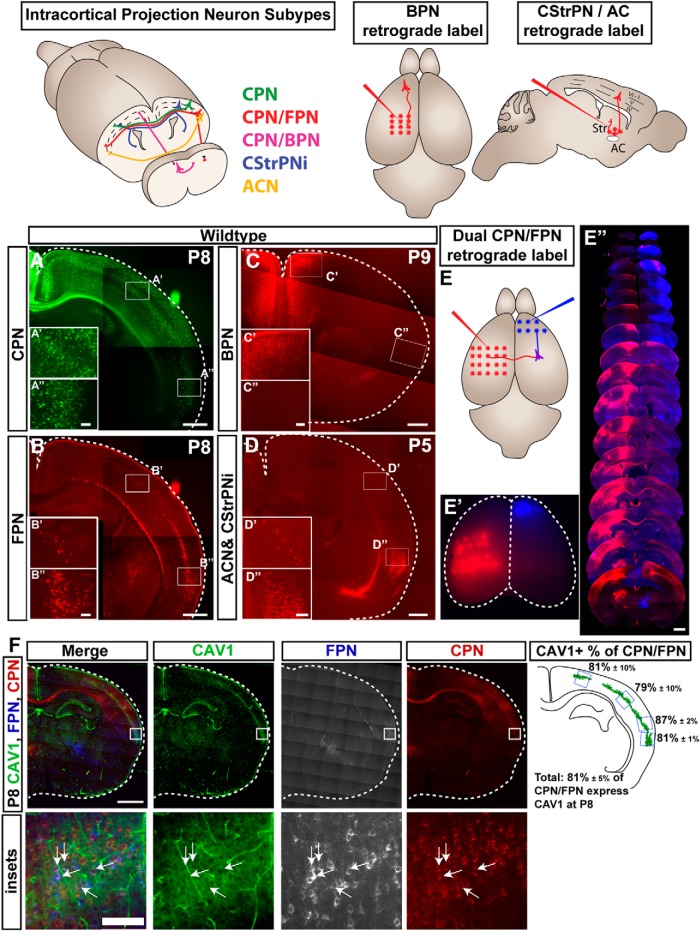
CAV1 is expressed by restricted subpopulations of neocortical neurons at P8, predominantly dual projecting CPN/FPN. Schematic representation of diverse populations of neocortical projection neurons with CPN projecting subsets, including CPN (green), CPN with a dual ipsilateral forward projection (CPN/FPN; red), CPN with a dual ipsilateral backward projection (CPN/BPN; magenta), intratelencephalic corticostriatal projection neurons (CStrPN_i_; blue), and anterior commissure projection neurons (ACN; yellow). ***A–D***, Retrograde labeling was performed for CPN (***A***; green), FPN (***B***; red), BPN (***C***; red), and CStrPN_i_/ACN (***D***; red). Both CPN and FPN are located entirely within the CAV1-expression domain, as are a subset of the mixed population of CStrPN_i_ and ACN laterally (in S2). BPN are restricted to the rostro-medial cortex, outside the CAV1-expression domain. ***E***, To identify dual-projecting CPN/FPN, we simultaneously injected CTB_647_ into the ipsilateral premotor cortex and CTB_555_ into the contralateral somatosensory cortex, as indicated by the schematic in ***E*** and wholemount image in ***E’***. ***E’’***, FPN are isolated predominantly in Layer V, with some in VIb (subplate) in largely lateral cortical locations, with caudo-lateral S2 expansion. CPN are located in Layers II/III, V, and VI. Dual projecting CPN/FPN are located in Layer Va in caudo-lateral cortex. ***F***, CAV1 is expressed by over 80% of dual projecting CPN/FPN. Dual projecting CPN/FPN were labeled as shown in ***E***, and the percentage of dual projecting CPN/FPN that express CAV1 was calculated for four medio-lateral regions, listed ±SEM (*n* = 4 brains). Scale bars: 500 μm (***A–D***), 100 μm (***A’–D’***, ***A’’–D’’***), 1 mm (***E’’***), 1 mm (***F***), and 100 μm (***F***, inset).

Development of CPN does not end when early progenitors are specified but, rather, includes acquisition of specific CPN subpopulation identities. Beyond the fact that CAV1 is highly expressed by CPN compared to CSMN overall, it is further quite specifically expressed in a very restricted pattern in neocortex. Because many uniquely projecting subpopulations of CPN reside in restricted cortical areas (in particular, within Layer V; Fame et al., 2011), and because the location of maximal CAV1 expression was reminiscent of the location of dual projecting neurons in mouse neocortex (Mitchell and Macklis, 2005), we investigated potential CAV1 expression by forward projecting neurons from somatosensory cortex to frontal areas (FPN), backward projecting neurons (BPN), CPN with contralateral striatal projections (CStrPN_i_), and commissural neurons with anterior commissure projections (ACN; Fig. 4*A–D*). The pattern of CAV1 expression closely resembles the restricted location of dual projecting FPN/CPN (Mitchell and Macklis, 2005), and partially overlaps with the location of CStrPN_i_ and ACN in the most lateral regions. Expression is highest at the time of axon and dendrite extension, and formation and stabilization of neuronal connections (P3; Fig. 2). The rostral location of BPN does not overlap with the CAV1-expression domain, indicating that CAV1 is not expressed by all dual projecting CPN populations; CAV1 expression is quite specific. Although some CStrPN_i_/ACN are located within the CAV1-expression domain laterally, the location of FPN/CPN closely overlaps with the CAV1-expression domain, suggesting that CAV1 might be specifically important for CPN/FPN development, connectivity, and/or function (Fig. 4).

To confirm more deeply at the individual neuron level whether CAV1 is expressed by the dual projecting FPN/CPN subpopulation, we retrogradely colabeled callosal- and frontal-projecting neurons by stereotaxic injection of CΤΒ conjugated to two different Alexa Fluor fluorophores into the contralateral somatosensory cortex and the ipsilateral premotor cortex, respectively (Fig. 4*E*,*F*). CAV1 overlaps with the entire domain of FPN, which also exist in other sensory modalities, including A1 (Mitchell and Macklis, 2005). We verified the proportion of CPN/FPN that express CAV1 in S1 and S2, since they represent the major population of CPN/FPN. We found that over 80% of dual projecting CPN/FPN are CAV1^+^, and only a small population of non-dual projecting cortical neurons expresses CAV1; CAV1 expression is thus highly restricted to and quite specific for this dual projecting population (Fig. 4*F*). Although the membranous location of CAV1 protein and the incomplete label generated by CTB injections preclude equivalent and reciprocal quantification of the percentage of CAV1^+^ cortical neurons that are dual-projecting, the domains map closely to the single neuron level.

### CAV1 is localized to CPN cell bodies and dendrites, and expression is not dependent on correct callosal connectivity

**Figure 5.**
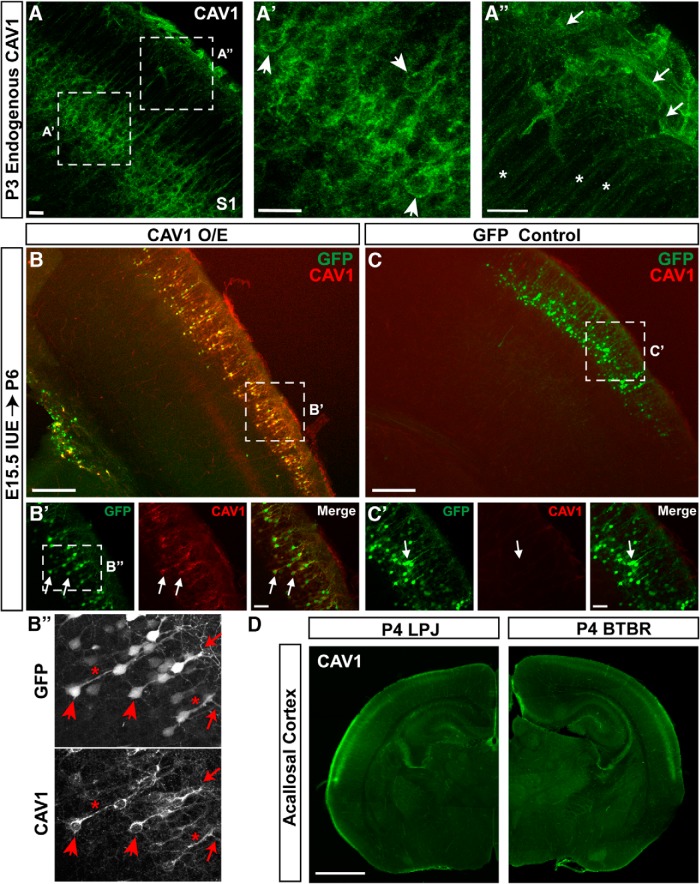
CAV1 is localized to neuronal cell bodies and dendrites at postnatal ages. ***A***, CAV1 is detected within distinct Layer Va neuronal cell bodies in S1 (arrowheads in ***A’***), extending throughout their apical dendrites (asterisks in ***A’’***) and their dendritic arborizations at the pial surface (arrows in ***A’’***). ***B***, ***C***, Exogenously expressing *Cav1* in superficial layer neurons of S1 by *in utero* electroporation at E15.5 does not disrupt migration or laminar location compared to a GFP-only control (***C***). ***B’***, ***C’***, Exogenous overexpression of *Cav1* results in CAV1 (red) protein localization that is similar to that of the endogenous protein (***A***), with CAV1 localized to the cell bodies and dendritic compartments of the neuronal plasma membrane of superficial layer neurons at P6. Unlike GFP (green), CAV1 is not detected throughout the soma and nucleus, or distributed throughout neuronal processes (dendrites and axons). ***B’’***, Higher magnification of ***B’***, comparing expression of CAV1 and GFP in soma (arrowheads), apical dendrites (asterisks), and dendritic tufts (arrows). ***D***, Correct CAV1 (green) expression is not dependent on formation of the CC at P4. Acallosal BTBR mice express CAV1 at comparable levels, and in an indistinguishable pattern, to their closely related callosal LPJ strain, indicating that CAV1 expression is not dependent on correct CPN connectivity. Scale bars: 20 μm (***A–A”***), 250 μm (***B***, ***C***), 50 μm (***B’***, ***C’***), and 1 mm (***D***).

CAV1 is a membrane-bound scaffolding protein, so its subcellular distribution might provide insight into its function(s) in specific neuronal subtypes. We investigated the subcellular localization of CAV1 in P3 CPN, and found it to be highly localized around the soma, extending throughout the apical dendrite and dendritic tuft (Fig. 5*A*). The same subcellular localization is observed at later stages of development (P7, data not shown). CAV1 is not highly detected in axons. This suggests potential roles for CAV1 in migration and/or dendrite function, although function at lower concentration in axons or growth cones is not excluded. Further, we generated a *Cav1-IRES-GFP* overexpression construct, and exogenously overexpressed *Cav1* in Layer II/III CPN of somatosensory cortex via *in utero* electroporation at E15.5. Superficial layer CPN, which do not normally express *Cav1*, show a similar cellular distribution of CAV1 to that of endogenous neurons, with strong localization around CPN somata and in apical dendrites at P6 (Fig. 5*B*). This is in direct contrast to overexpressed GFP, which is evenly distributed throughout the neuronal soma, dendritic arbor, and axons, in which it is readily detectable (Fig. 5*C*,*B’’* ).

Since CAV1 is known to interact with striatins for correct signal transduction at synapses (Gaillard et al., 2001), and with multiple neurotransmitter receptors (Boulware et al., 2007; Takayasu et al., 2010), we considered that CAV1 expression might be regulated by neuronal activity. However, based on the early neonatal expression of CAV1, we hypothesized that final connectivity is not needed for CAV1 expression. To investigate whether CPN connectivity is required for postnatal CAV1 expression, we investigated whether CAV1 expression is altered in acallosal BTBR mice (Wahlsten et al., 2003). The BTBR mouse strain exhibits otherwise largely normal cortical development, but no axons extend across the CC. Although they perform quite well on physical coordination tasks, they display behavioral abnormalities. Additionally, these mice exhibit a reduced hippocampal commissure, accompanied by improper wiring reflected by tangles of axons known as probst bundles. These experiments reveal that CAV1 expression is independent of correct callosal connectivity, and CAV1 is expressed normally in P4 BTBR neocortex (Fig. 5*D*). Taken together, these results indicate that, although CAV1 had been previously shown to act at synapses (Gaillard et al., 2001), its expression by developing CPN is not dependent on correct connectivity.

### *Cav1* function is not necessary for dual-projecting FPN/CPN to reach their axonal targets

**Figure 6.**
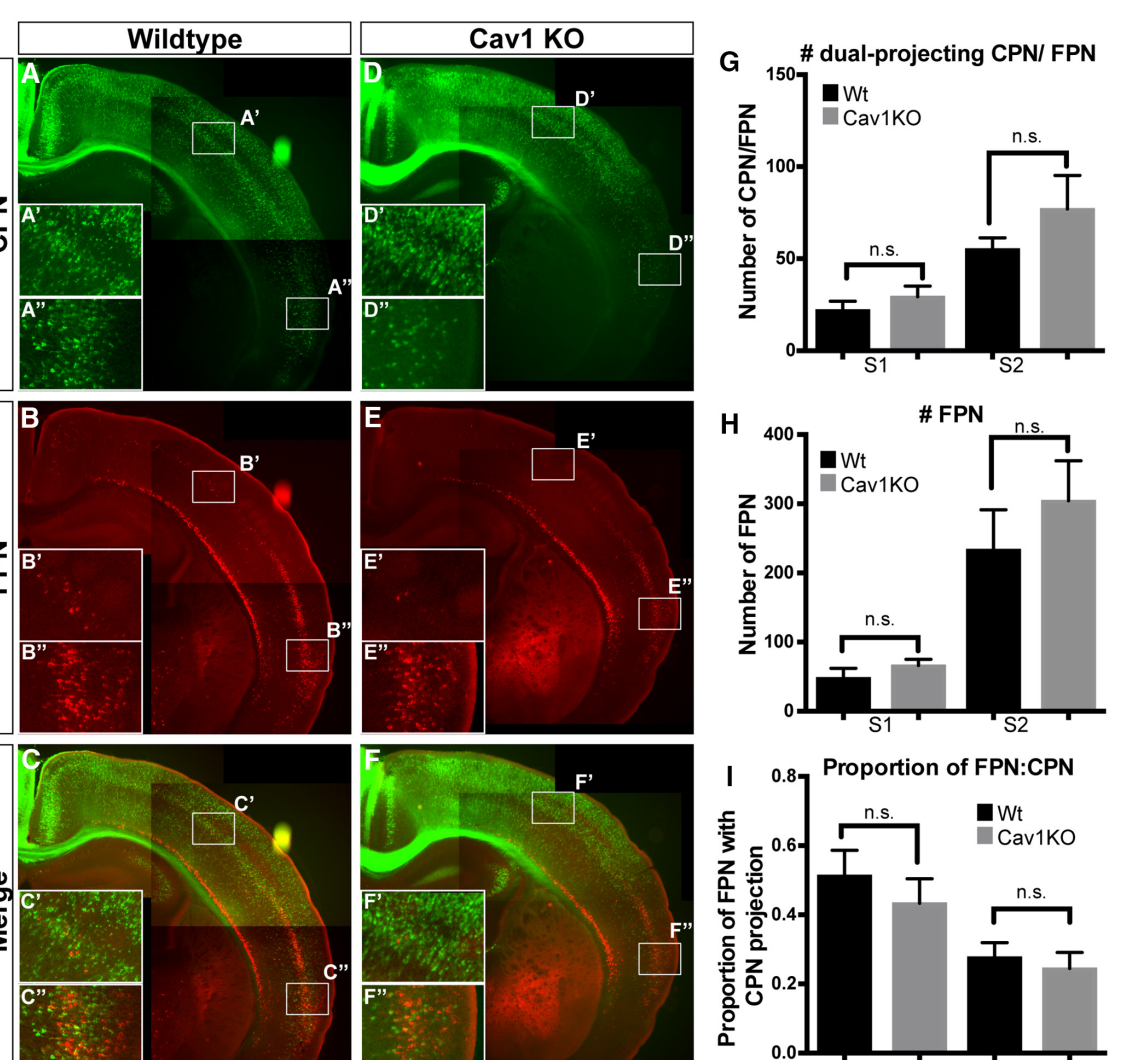
Loss of *Cav1* function does not disrupt formation of dual projecting CPN/FPN axonal projections at P8. ***A–F***, CPN (green) were retrogradely labeled from contralateral somatosensory cortex, and FPN (red) from ipsilateral premotor cortex in *Cav1*-null mice (***D–F***; *n* = 5) and WT littermates (***A–C***; *n* = 5). ***A***, ***D***, CPN; ***B***, ***E***, FPN; and ***C***, ***F***, CPN/FPN form in the absence of *Cav1* function. ***G***, ***H***, The number of dual projecting CPN/FPN (***G***) and total FPN (***H***) were counted for two medio-lateral regions (S1 and S2). There is no significant difference between the two genotypes (*n* = 5 WT and *n* = 5 *Cav1*-nulls). ***I***, The percentage of total labeled FPN that have a dual callosal projection was also calculated for two medio-lateral regions (S1 and S2). There is no significant difference between the two genotypes (*n* = 5 WT and 5 *Cav1*-null). Error bars denote SEM. FPN, ipsilateral FPN. Scale bars: 500 μm (***A–F***) and 100 μm (***A’–F’***, ***A’’–F’’***).

Because CAV1 is expressed by the overwhelming majority of dual-projecting FPN/CPN at P8, and most highly at P3, and although it not enriched in axons, we investigated whether *Cav1* might be necessary for the correct maturation and/or maintenance of this specific subpopulation of CPN. However, because of its subcellular localization to the somato-dendritic compartments, we hypothesized that CAV1 would not be necessary for axonal guidance or targeting. We first investigated whether axons of FPN/CPN initially reach their targets correctly in the absence of *Cav1* function, which would indicate that *Cav1* is not necessary for axonal extension, pathfinding, or establishment of CPN connectivity. We retrogradely labeled FPN/CPN in both *Cav1*-null mice and their WT littermates at P6, as described previously, and examined them at P8 (Fig. 6). At this relatively early time (P8), when CPN exuberance is most pronounced, there is no difference in the overall number of FPN/CPN between *Cav1*-null and WT mice (Fig. 6*G*), including within the secondary somatosensory cortical area (S2), where both CAV1 expression and the abundance of FPN/CPN change dramatically. Thus, as predicted by its subcellular localization, *Cav1* function is not required for CPN/FPN to extend dual-projecting axons to specific targets. Since CAV1 is localized around neuronal cell bodies and dendritic trees, but not substantially in axons, it is not surprising that FPN/CPN reach their targets at P8, further supporting somato-dendritic-specific function for *Cav1*.

CAV1 expression and localization in CPN/FPN during early neuronal maturation suggests subtype-specific function(s) during processes such as migration, neurite outgrowth, and branching. The previously known scaffolding roles of CAV1 at synapses, and its interactions with neurotransmitter receptors and Rac1 in other systems, suggest that CAV1 might influence neuronal activity through axonal/dendritic connectivity and/or function. Our results identify highly specific neocortical CAV1 expression in the dendritic compartment of dual projecting CPN/FPN, and further identifies both that CAV1 expression is not dependent on axonal connectivity, and that correct axonal targeting of CPN/FPN is independent of CAV1.

## Discussion

Identifying molecular markers and determinants of distinct subsets of CPN of the cerebral cortex will enable specific and rigorous investigation of distinct and unique subpopulations potentially critical for information integration and associative connectivity. CPN reside in cortical Layers II/III, V, and VI and all extend axons to homotopic targets in the contralateral hemisphere. Some CPN subpopulations extend second (or more) axons to distinct targets including frontal or caudal ispsilateral neocortex, or even subcortically into ipsi- or contralateral striatum (Mitchell and Macklis, 2005; Cederquist et al., 2013; Sohur et al., 2014). Increasingly specific markers and determinants will also potentially enable in-depth functional analysis of these determinants themselves through development, to gain insight into their roles in establishing precise connectivity that endows specific CPN subpopulations with critical roles in neocortical information transfer, correlation, and integration.

Here, we identify that CAV1, a lipid raft scaffolding protein enriched in CPN over CSMN (Molyneaux et al., 2009), is expressed in a restricted fashion in the developing neocortex, and is expressed by over 80% of a unique, interesting, and likely functionally special CPN subpopulation – dual projecting CPN extending axons both contralaterally and to ipsilateral frontal areas (CPN/FPN). Comparative microarray analyses have identified molecular markers and determinants of CPN subpopulations, and subsequent evaluation of these and other data reveal molecular diversity within the broad population of CPN (Molyneaux et al., 2009; Fame et al., 2016a,b). This diversity includes not only laminar and areal subpopulations, but also unique expression patterns that reflect subpopulations of CPN with distinct and sometimes multi-target axonal projections. CAV1 is a first studied example of such multi-target CPN identifiers; interestingly, it is localized to CPN cell bodies and dendrites, but is not detected in axons. The developmental temporal expression of *Cav1* coincides with the intermediate time period of CPN development, including low-level expression during neuronal migration, and highest levels of expression from P3 to P6 in mice, when CPN are extending and refining axonal and dendritic processes. Together, these results suggest functions for *Cav1* in postmitotic establishment of innervation and/or pruning of connections.

CAV1 has a unique, areally-restricted expression pattern within CPN of Layer Va; it is coexpressed with BHLHB5 in S1, but excluded from the LMO4-expressing M1. The reciprocal expression of LMO4 and BHLHB5 in M1 and S1, respectfully, is important for establishing these areal identities (Joshi et al., 2008b; Cederquist et al., 2013). Further, *Lmo4* loss-of-function disrupts the development of dual-projecting CPN/BPN, whose cell bodies reside in the rostral motor cortex, and which send axons callosally and caudally (Cederquist et al., 2013). Thus, it is interesting to speculate that *Bhlhb5* loss-of-function might similarly disrupt development of the CAV1^+^ dual projecting CPN/FPN.

Here, we further report that *Cav1* function is not necessary for specification and early development of dual projecting CPN/FPN, investigated by precise dual retrograde labeling approaches in *Cav1*-null neocortex. These results are consistent with *Cav1’s* later developmental timing of expression, and absence of subcellular localization to axons. Because these CPN/FPN projections continue to prune and establish precise connectivity until P21 (Mitchell and Macklis, 2005), future studies might employ the same or related dual labeling approaches progressively to or beyond P21 to investigate potential changes in axonal pruning that might result from potentially improper dendritic arborization, dendritic synaptogenesis, and/or axonal target-finding. If there is a change in the number of CPN/FPN in *Cav1*-null mice at P21, comparison between the number of CPN/FPN detected with retrograde labeling at P21 to the number detected at P21 from retrograde labeling performed at P8, for example, would be able to distinguish between axonal pruning of one or both projections, versus loss of these neurons altogether.

Because of CAV1’s known interaction with neurotransmitter receptors (Lai et al., 2004; Boulware et al., 2007; Takayasu et al., 2010), dendritic spine signaling scaffolds (Gaillard et al., 2001), and synaptosome components (Bilderback et al., 1997; Bilderback et al., 1999; Braun and Madison, 2000) function(s) of CAV1 in CPN/FPN might only be elucidated optimally through electrophysiological functional analysis of these neurons lacking their endogenous *Cav1* function in *in vivo* circuits. We find that *Cav1* expression is not dependent on correct CPN connectivity, by investigating CAV1 expression in the acallosal BTBR and *Fezf2* ^−/−^ mouse lines. However, *Cav1* might still be critical for CPN/FPN neuronal activity, since neuronal activity is often tightly tied to axonal and dendritic connectivity (Wang et al., 2007; Cubelos et al., 2010; Rodríguez-Tornos et al., 2016), especially establishment and maintenance. Studies examining P21 CPN/FPN axonal and dendritic morphologies with loss- or gain-of-function of *Cav1* might reveal CAV1 function(s) in CPN/FPN activity.

We also identify that CAV1 is highly concentrated in cell bodies and apical dendrites of CPN/FPN. Interestingly, CAV1 is also localized to the cell bodies and dendrites of a subpopulation of pyramidal cortical neurons in primates (Fame et al., 2016a), although older studies suggest that CPN/FPN might not be maintained in primates but, rather, might be pruned (Schwartz and Goldman-Rakic, 1982, 1984; Andersen et al., 1985; Johnson et al., 1989; Meissirel et al., 1991). Regardless of whether there are large or relatively rare primate CPN/FPN subpopulations, some unique properties of this subpopulation are likely conserved between mice and primates. Because of the identified conserved and specific subcellular localization of CAV1, future work might examine dendritic development and maintenance in *Cav1*-null CPN/FPN in comparison to WT. Together, these studies could provide important insight into the development of this unique dual projecting population of CPN that likely function in a variety of complex and critical processes, integrating cortical information between hemispheres and from primary sensorimotor areas to premotor cortices.

Future studies could investigate whether CAV1 directly interacts in CPN with a subset of previously identified CAV1 binding partners (particularly those shown to act in neuronal-relevant processes), and whether CAV1 functions in the development of defined subpopulations of CPN through these interactions. CAV1 is known to have a number of protein interactors from studies in other systems. One particularly compelling potential interacting protein for first analysis in CPN/FPN is Rac1, given that it has been shown in other systems to play roles in important neuronal functions relating to cytoskeletal dynamics and focal adhesion, such as neurite growth, adhesion, and migration (Beardsley et al., 2005; Kang et al., 2006; Joshi et al., 2008a). In particular, Rac1 is required for midline crossing of CPN (Chen et al., 2007; Kassai et al., 2008), and Rac1, recruited by CDKL5, can regulate neuronal migration and dendritic arborization of some CPN (Chen et al., 2010). Identification of neocortical binding partners of CAV1 could provide valuable insight into mechanisms of CPN subtype development/refinement through complex networks of discrete protein-protein interactions, with potential implications for subtypes of autism spectrum disorders (ASD) and/or schizophrenia. The emerging “cortical connectivity/synaptogenic hypothesis” of ASD suggests that such change(s) caused by *Cav1* dysfunction might contribute to ASD and related behavioral phenotypes.

Interestingly, human *Cav1* is located at 7q31.1, part of autism-linked locus 9 (Auts9), immediately upstream of *MET*, which shows direct pretranscription start-site mutations associated with ASD (Campbell et al., 2006). It might be that *Cav1* is also directly relevant in ASD, and might potentially contribute to some of the Auts9 linkage to the disorders, perhaps via CPN. *Cav1* is also close genomically to *Foxp2* in Auts9, which was initially suspected by some to underlie ASD language defects, but was later shown to not be causal of the Auts9 ASD linkage (Newbury et al., 2002). Other potential gene linkages in this locus (NRCAM and ST7) are relatively weak, indicating that the linkage must be accounted for, at least in part, by other Auts9 genes, potentially partially by *Cav1*. CAV1 has also been implicated as a potential target for schizophrenia therapy due to its interaction with DISC1, and its ability to modulate DISC1 expression in neurons (Kassan et al., 2017).

The data we present here are, to our knowledge, the first identification of a potential molecular control over a unique and specialized dual projecting subpopulation of CPN, CPN/FPN. While CAV1 might likely function as a specific developmental and/or functional regulator in CPN/FPN, its specificity within this population also immediately provides a molecular marker. This will allow future studies to identify, genetically target, and/or purify this population to investigate and potentially discover additional potential upstream controls over CPN/FPN development. The defining properties of this CPN/FPN subpopulation, likely critical for “feed forward” information integration, are yet to be well understood, but analysis of *Cav1* expression and function identifies and characterizes a first molecular marker and determinant of this unique associative and integrative neocortical projection neuron population.

*Note Added in Proof:* The first names of the first and last author were accidentally switched and incorrectly listed in the Early Release version published January 8, 2018. The author names have now been corrected.

## Synthesis

Reviewing Editor: Menno Witter, Norwegian University of Science and Technology Decisions are customarily a result of the Reviewing Editor and the peer reviewers coming together and discussing their recommendations until a consensus is reached. When revisions are invited, a fact-based synthesis statement explaining their decision and outlining what is needed to prepare a revision will be listed below. The following reviewer(s) agreed to reveal their identity: Linda Richards, Dhananjay Huilgol.

Advances

Callosal projection neurons (CPN) have so far been defined based on axon projections only. Even though there are multiple subcategories depending on where the neurons project, they have not been distinguished in terms of molecular markers. This manuscript provides the first report of characterizing one such CPN type, which will help study these neurons both in terms of development and function.

General.

This manuscript provides the first report of a molecular marker defining a specific category of callosal projection neurons (CPNs), restricted in terms of their laminar location and cortical areas. The authors characterize the localization of the molecule, Caveolin 1 (CAV1) to the cell body and dendrites and analyze the knockout to understand the developmental stage at which this molecule might be necessary. They report that CAV1 is not required for callosal early axonal specification and that its expression is not dependent on the formation of the corpus callosum. Based on these results, the authors suggest that CAV1 might have a somato-dendritic specific function, controlling the establishment and maintenance of dendritic arbors in this sub-type of CPNs. The data provide the field with an entry point to study the development and function of the CPN/FPNs and leads way for characterizing molecular signatures of other subtypes of CPNs.

A strength of this study is that the authors explore the function of CAV1 using retrograde tracer injections in wild type and Cav1 KO mice, in utero electroporation of a Cav1 overexpression construct, and using a BTBR mouse model of agenesis of the corpus callosum. Despite the fact that the authors did not perform functional experiments of the role of Cav1 in dendritic development and maturation in CPNs, the authors present a thorough investigation into the expression of CAV1. Overall, this study is interesting and relevant for the field as it contributes to our limited understanding on the heterogeneity of CPNs.

The research questions presented in the paper are clear and the data supports the authors' main conclusions that CAV1 is not required for connectivity or specification of the CPN subpopulations and that this protein can be used as a unique identifier for a multitarget CPN population.

The manuscript is overall well-written but the following comments should be addressed to improve the manuscript:

1. In the Introduction on Page 3, authors mention about molecular controls defining/regulating CPNs that are beginning to be elucidated. The authors should elaborate on some of these molecules as well as refer to the specific studies that have provided an advance in this direction.

2. How early does the expression of CAV1 begin in the mouse neocortex? The authors refer to Li et al., 2011 which show the expression of CAV1 in Rat E15-16 progenitors (approximately, mouse E13-14), whereas E18.5 is the earliest time point the authors show in Figure 2. This will be interesting to understand the role of CAV1 in neural progenitors. Furthermore, future studies can identify interacting partners that provide redundancy such that the development of these specific CPN/FPNs is unaffected in CAV1 KO (Figure 6).

3. Page 14: Related to figure 3, the authors describe a sub-population of non-SCPNs that express CTIP2 and CAV1. How do they conclude that these neurons do not project subcerebrally? Is it based on their location? The authors suggest that they may be a subpopulation of neurons with transient developmental spinal projection that is later lost. Is it possible that these neurons are actually interneurons instead (Nikouei et al., 2016, Journal of Chemical Neuroanatomy)?

4. In Figure 3A, the authors show exclusion of CAV1 versus Lmo4 expression in the cortex. Lmo4 and Bhlhb5 have opposite expression patterns in the cortex. In addition, Bhlhb5 is necessary for areal identities (Joshi et al., 2008). It will be interesting to know the authors' perspective on the development of CAV1-expressing CPN/FPNs in Bhlhb5 knockout in the Discussion. This will bring other known molecular players in context of CPN/FPN development.

5. In Figure 3, the authors convincingly show that CAV1(+) neurons are excluded from the majority of Ctip2(+) cells. They also need to show co-localization with Layer Va markers such as PlexinD1, or ER81 (broad Layer V marker).

6. Is the expression of CAV1 specific to S1 and caudo-lateral S2? How about other primary sensory cortices such as V1 and A1? If there is expression in V1 or A1, where do the CAV1(+) FPNs project?

Minor comments:

Main text:

7. There are some areas in the main text where the sentences are long and complex and could be made more concise, for example, the last sentence of the abstract and the initial part of the discussion.

8. Retrograde labelling methodology

- For each injection type, the authors should describe the methodology with further detail, describing how they determined the region of interest for each injection. For example, were stereotaxic coordinates used to locate these areas?

- How was the anterior commissure targeted without non-specific labelling, given its small size and ventral location? For example, was the anterior commissure targeted directly at the midline.

The authors write that they performed 25 injection sites to label CPNs with projections to the contralateral neocortex. Ipsilateral projection neurons were labelled with 5 injection sites (even though the schematic shows 7 injection sites in the premotor cortex). “Backward” projecting neurons (change this to “caudally projecting neurons”) were labelled with 12 injection sites, and the number of injections in the anterior commissure and striatum are not mentioned.

9. In Results on Page 13, the sentence “...CAV1 expression is unchanged in Fezf2 loss-of-function neocortex, in which no subcerebral neurons are specified (Molyneaux et al., 2005), confirming that CAV1 is excluded from CSMN (Figure 1C)” should be changed to Figure 1D, E.

10. In Results, Page 14, the Figure reference after the sentence “.... consistent with the dominant birthdate ranges for neocortical neurons residing in layer V (Figure 3D,E)” should be changed to (Figure 3,D-F).

11. The age on Figure 4B is mentioned as P8, whereas it is written as P6 in the text on Page 16 (Results).

12. Please correct the figure reference on Page 17 after the sentence “....correct callosal connectivity, and CAV1 is expressed normally in P4 BTBR neocortex (Figure 4D)”. It should be Figure 5C.

Figures, general comments:

The authors should delineate and label the cortical layers and the origin of the insets in all the histological figures in order to more easily demonstrate where the layer makers are expressed. Moreover, the age of experimental animals should be consistent in the figures, in the legends and in the manuscript, as often these ages did not coincide.

Fig.1:

- The schematics are nice but they should not replace images of the injection sites. These should be included as evidence of the injection site.

- How many animals were injected? It would help to add some quantitative data of the proportion of CPN and SCPN retrogradely labelled cells express CAV1.

- Panel B: the images are too small and insets are needed to show the location of the images in panel C. In addition, to match the high magnification images in C, it would be good to show a whole section for the CPN tracing in B.

- The authors should clarify that the injection was done only at P1 and not between P1 and P3.

Fig. 2:

- The age of the experimental animals (P6 in the figure and P7 in the figure legend) does not match.

- Panel B: Include DAPI staining to make the figure clearer. Also, the images are pixelated particularly in the first panel. Please use an image with better resolution.

Fig. 3:

- Panel B and C: Clarify on the figure which age is represented. It would be good to have a higher magnification image to see the co-expression of CAV1 and CTIP2.

- Panel F: what is the n for quantification analysis?

Fig. 4:

In the legend as well as the manuscript, the authors write “the only specific population of cortico-cortical projection neurons located entirely within the CAV1 expression domain are FPN. However, a portion of the mixed population of CStrPNi and ACN also overlaps with the CAV1 expression domain laterally”. Provide a panel showing this overlap, or have the CAV1 staining shown in all the panels with the retrogradely labelled cell bodies in the different injections performed.

The authors also state that “to identify dual-projecting CPN/FPN, we simultaneously injected CTB-647 into the ipsilateral premotor cortex and contralateral somatosensory cortex...” From panel E, it is shown that they injected a different CTB (Alexa555 conjugated) in the somatosensory cortex.

- Panels A-D: Include DAPI staining for morphology.

- Panels C-D: Is there a reason for different ages of injection for BPN and ACN?

- Panel E: It would be helpful to include a schematic of the CTB injection sites for BPN, ACN and CStrPNi, similar to the CPN and FPN injections already shown in E.

- Panel E': It is not very clear, maybe drawing the border of the brain section might help to understand the localisation of the injection site. And given the high number of injections per animal, it would be good to have an idea of the survival rate in the methods.

- Panel E“: The series of coronal images here is well presented, however, it is difficult to distinguish the cortical layers as described in legend. Some representative sections could perhaps be enlarged to show this.

- Panel F: What is the n for quantification analysis? Increase the size of the inset images and the arrows used in them.

Fig.5:

- Panel A: It is not clear where these images are taken from in the cortex. It would also help to provide higher magnification images to show specific cellular components, particularly with respect to the localisation of CAV1 expression.

- Panel B: there is no context of cortical location. The authors describe the cellular location of CAV1 protein in neurons of pups electroporated at E15.5 and collected at P8, but in the manuscript and in the figure legend the authors state that the pups were collected at P6? It would also help to show higher magnification images (as per panel A). This panel is also referred to as Figure 4B in the text.

- Panel C: is referred to as panel D in the manuscript.

In Figure 5A, CAV1 is shown to be localized around the soma, apical dendrite and dendritic tuft at P3. Does the expression of CAV1 change over development, for example, what is the subcellular localization of CAV1 at P8? This would suggest possible different roles of CAV1 at different developmental stages.

Fig.6:

- Table describing t-test results on page 11: Do the bottom two lines refer Fig 6I and not 6H?

## Author Response

We thank the reviewers and editor for their detailed review, and insightful and constructive suggestions regarding our manuscript “Caveolin 1 identifies a specific subpopulation of cerebral cortex callosal projection neurons (CPN) including dual projecting cortical callosal/frontal projection neurons (CPN/FPN)”. We have now revised the manuscript in response to each review, not only with additional data, but also with textual clarification of our results and conclusions, as outlined below. All changes within the text are indicated with blue font. The reviews and these revisions make the manuscript stronger; we hope and trust that the editors and reviewers will agree, and that the revised manuscript will now be found acceptable for publication in eNeuro.

1. In the Introduction on Page 3, authors mention about molecular controls defining/regulating CPNs that are beginning to be elucidated. The authors should elaborate on some of these molecules as well as refer to the specific studies that have provided an advance in this direction.

- We thank the reviewers for this helpful suggestion, and we have now added a brief discussion of several such examples on page three of the Introduction, including Satb2, Cux1 and Cux2, Cited2, and Ctip1.

2. How early does the expression of CAV1 begin in the mouse neocortex? The authors refer to Li et al., 2011 which show the expression of CAV1 in Rat E15-16 progenitors (approximately, mouse E13-14), whereas E18.5 is the earliest time point the authors show in Figure 2. This will be interesting to understand the role of CAV1 in neural progenitors. Furthermore, future studies can identify interacting partners that provide redundancy such that the development of these specific CPN/FPNs is unaffected in CAV1 KO (Figure 6).

- We examined expression of Cav1 by in situ hybridization at earlier embryonic timepoints, and have now included images from E13.5 and E15.5 to Figure 2. We do not detect Cav1 expression by cortical progenitors in mouse at any age. At 13.5, Cav1 is expressed in the presumptive developing hippocampus, but is absent from the cortex. Caudolateral expression of Cav1 is apparent in the cortical plate by E15.5, in a similar pattern to that observed at later developmental timepoints, but Cav1 is not expressed by VZ or SVZ progenitors. Thus, the role of Cav1 in cortical development (at least in mouse) appears to be restricted to postmitotic neurons.

3. Page 14: Related to figure 3, the authors describe a sub-population of non-SCPNs that express CTIP2 and CAV1. How do they conclude that these neurons do not project subcerebrally? Is it based on their location? The authors suggest that they may be a subpopulation of neurons with transient developmental spinal projection that is later lost. Is it possible that these neurons are actually interneurons instead (Nikouei et al., 2016, Journal of Chemical Neuroanatomy)?

- We thank the reviewer for highlighting this lack of clarity in our description of this population. Although we did not directly examine expression of CAV1 by molecularly-identified interneurons, these neurons appear to be pyramidal projection neurons based on their size and morphology. We concluded that they do not project subcerebrally based on the lack of overlap between CAV1 expression and SCPN demonstrated in Figure 1. We have now clarified both of these points in the Results text.

4. In Figure 3A, the authors show exclusion of CAV1 versus Lmo4 expression in the cortex. Lmo4 and Bhlhb5 have opposite expression patterns in the cortex. In addition, Bhlhb5 is necessary for areal identities (Joshi et al., 2008). It will be interesting to know the authors' perspective on the development of CAV1-expressing CPN/FPNs in Bhlhb5 knockout in the Discussion. This will bring other known molecular players in context of CPN/FPN development.

- This is an interesting suggestion. We previously demonstrated that Lmo4 loss-of-function disrupts the development of dual-projecting CPN/BPN, whose cell bodies reside in the rostral motor cortex, and send axons callosally and caudally (Cederquist et al, J Neurosci 2013). Thus, it is conceivable that Bhlhb5 loss-of-function could similarly disrupt CPN/FPN development. We have now added this speculation to the Discussion, in addition to examining co-expression of CAV1 and BHLHB5 (Figure 3).

5. In Figure 3, the authors convincingly show that CAV1(+) neurons are excluded from the majority of Ctip2(+) cells. They also need to show co-localization with Layer Va markers such as PlexinD1, or ER81 (broad Layer V marker).

- We have now added additional co-expression of CAV1 and the CPN molecular control SATB2 to Figure 1 to demonstrate the CPN-specific expression of CAV1, complementing the retrograde labels. To further demonstrate that CAV1 is expressed in layer 5, we now show in Figure 3 that CAV1 expression is located below layer IV in S1 by labeling the barrels with 5-HT, and we demonstrate that it is co-expressed with Bhlhb5, which is expressed in layer V, but not layer V1. Unfortunately, we were not able to directly investigate co-expression of CAV1 with the layer V marker PlexinD1 due to incompatibilities of the primary antibodies.

6. Is the expression of CAV1 specific to S1 and caudo-lateral S2? How about other primary sensory cortices such as V1 and A1? If there is expression in V1 or A1, where do the CAV1(+) FPNs project?

- We thank the reviewers for these interesting questions. Expression of CAV1 appears to extend into presumptive A1, although we do not detect it more medially in V1 at the ages examined. Association projections to the frontal cortex integrate input from several sensory modalities, including somatosensory, auditory, and posterior parietal cortical areas (Barbaresi et al., 1989; Barbas and Pandya, 1989; Barbas, 1995; Carmichael and Price, 1995; Reep et al., 1996; Mitchell and Cauller, 2001). CAV1 overlaps with their entire domain. We verified the proportion of CPN/FPN with CAV1 in S1 and S2, since they represent the major populations of CPN/FPN, but these dual projection neurons also exist in A1 (Mitchell et al., 2005). We have clarified this point within the Results text for Figure 4. This would be an interesting point for future investigations.

Main text:

7. There are some areas in the main text where the sentences are long and complex and could be made more concise, for example, the last sentence of the abstract and the initial part of the discussion.

- We have edited to clarify our sentences throughout, in addition to simplifying the last sentence of the abstract and the opening to the discussion.

8. Retrograde labelling methodology

- For each injection type, the authors should describe the methodology with further detail, describing how they determined the region of interest for each injection. For example, were stereotaxic coordinates used to locate these areas?

- How was the anterior commissure targeted without non-specific labelling, given its small size and ventral location? For example, was the anterior commissure targeted directly at the midline.

The authors write that they performed 25 injection sites to label CPNs with projections to the contralateral neocortex. Ipsilateral projection neurons were labelled with 5 injection sites (even though the schematic shows 7 injection sites in the premotor cortex). “Backward” projecting neurons (change this to “caudally projecting neurons”) were labelled with 12 injection sites, and the number of injections in the anterior commissure and striatum are not mentioned.

- We have added substantial additional details to the Methods to clarify our retrograde labelling methodology. This includes stereotaxic coordinates, and clarification of the number of injection sites, injection volumes, and the developmental ages for each targeted neuronal population.

- Respectfully, we have maintained the nomenclature of “Backward” projecting neurons, both for consistency with our previous publication investigating this population (Cederquist et al, Journal of Neuroscience 2013), and to enable an abbreviation of “BPN” rather than a second “CPN” or “CaudPN”.

9. In Results on Page 13, the sentence “...CAV1 expression is unchanged in Fezf2 loss-of-function neocortex, in which no subcerebral neurons are specified (Molyneaux et al., 2005), confirming that CAV1 is excluded from CSMN (Figure 1C)” should be changed to Figure 1D, E.

10. In Results, Page 14, the Figure reference after the sentence “.... consistent with the dominant birthdate ranges for neocortical neurons residing in layer V (Figure 3D,E)” should be changed to (Figure 3,D-F).

11. The age on Figure 4B is mentioned as P8, whereas it is written as P6 in the text on Page 16 (Results).

12. Please correct the figure reference on Page 17 after the sentence “....correct callosal connectivity, and CAV1 is expressed normally in P4 BTBR neocortex (Figure 4D)”. It should be Figure 5C.

- We apologize for all of these inadvertent errors (holdovers from previous drafts / organizations of the manuscript), and we have corrected all.

Figures, general comments:

The authors should delineate and label the cortical layers and the origin of the insets in all the histological figures in order to more easily demonstrate where the layer makers are expressed. Moreover, the age of experimental animals should be consistent in the figures, in the legends and in the manuscript, as often these ages did not coincide.

Fig.1:

- The schematics are nice but they should not replace images of the injection sites. These should be included as evidence of the injection site.

- How many animals were injected? It would help to add some quantitative data of the proportion of CPN and SCPN retrogradely labelled cells express CAV1.

- Panel B: the images are too small and insets are needed to show the location of the images in panel C. In addition, to match the high magnification images in C, it would be good to show a whole section for the CPN tracing in B.

- The authors should clarify that the injection was done only at P1 and not between P1 and P3.

- We thank the reviewers for the helpful suggestion to add images of the injection sites. We have now included example images for both CPN and SCPN labeling, with wholemount images to show the injection points (dorsal view for CPN, ventral for SCPN), and coronal sections to show the distribution of labeled neurons in both low and high magnification.

- A minimum of 6 animals were examined by retrograde labeling across multiple litters.

- We do provide quantification of the percent of CPN/FPN (a regionally- and laminarly-restricted subpopulation of CPN) that express CAV1 in Figure 4. However, we did not quantify the percentage of SCPN that express CAV1 since SCPN do not overlap with the CAV1 expression domain, and examples of overlap are extremely rare. CPN are a broad population of neurons located throughout the cortex in multiple lamina, and our retrograde labeling approach cannot label the entire population. Thus, it is not feasible to reliably quantify the percent of all CPN that express CAV1.

- We have now included larger high magnification inset images, and a larger low magnification image of the merged channels to more clearly show the region of the inset.

- We have clarified that the retrograde labeling injections were performed at P1, and the analysis was performed at P3.

Fig. 2:

- The age of the experimental animals (P6 in the figure and P7 in the figure legend) does not match.

- Panel B: Include DAPI staining to make the figure clearer. Also, the images are pixelated particularly in the first panel. Please use an image with better resolution.

- We have now clarified this inconsistency in the age of the animals (P6).

- We apologize that the resolution appeared poor in the final PDF. We have reassembled the figure in Adobe Illustrator from the original panels, and find that the resolution is now improved.

- We have additionally added demarcation of the cortical lamina to identify the regions of expression, which enhances the clarity of the figure even beyond what a counterstain (such as DAPI) might provide.

- In addition, we have added in situ hybridization images (A) of earlier embryonic timepoints, as discussed in Major Point 2.

Fig. 3:

- Panel B and C: Clarify on the figure which age is represented. It would be good to have a higher magnification image to see the co-expression of CAV1 and CTIP2.

- Panel F: what is the n for quantification analysis?

- We have added the age of analysis (P1) to B and C, in addition to high magnification insets (indicated by boxes) to better demonstrate the co-expression of CTIP2 and CAV1. In addition, we have added cortical lamina demarcations to A' to enhance the clarity.

- To make room, we have removed the quantification in Panel F. The birthdating of cortical lamina is already well-established, and we agree that providing larger images of co-localization with CAV1 is more informative.

- As outlined above, we have added data on co-localization of CAV1 with lamina-specific markers to this figure as well.

Fig. 4:

In the legend as well as the manuscript, the authors write “the only specific population of cortico-cortical projection neurons located entirely within the CAV1 expression domain are FPN. However, a portion of the mixed population of CStrPNi and ACN also overlaps with the CAV1 expression domain laterally”. Provide a panel showing this overlap, or have the CAV1 staining shown in all the panels with the retrogradely labelled cell bodies in the different injections performed.

The authors also state that “to identify dual-projecting CPN/FPN, we simultaneously injected CTB-647 into the ipsilateral premotor cortex and contralateral somatosensory cortex...” From panel E, it is shown that they injected a different CTB (Alexa555 conjugated) in the somatosensory cortex.

- Panels A-D: Include DAPI staining for morphology.

- Panels C-D: Is there a reason for different ages of injection for BPN and ACN?

- Panel E: It would be helpful to include a schematic of the CTB injection sites for BPN, ACN and CStrPNi, similar to the CPN and FPN injections already shown in E.

- Panel E': It is not very clear, maybe drawing the border of the brain section might help to understand the localisation of the injection site. And given the high number of injections per animal, it would be good to have an idea of the survival rate in the methods.

- Panel E“: The series of coronal images here is well presented, however, it is difficult to distinguish the cortical layers as described in legend. Some representative sections could perhaps be enlarged to show this.

- Panel F: What is the n for quantification analysis? Increase the size of the inset images and the arrows used in them.

- We thank the reviewers for highlighting this lack of clarity with respect to our identification of dual projecting CPN populations that might express CAV1. Our intention was to first identify the overall location of these subpopulations (A-D) in comparison to the expression domain of CAV1 to determine which populations fall within the expression domain of CAV1. Based on this analysis, we were able to narrow our focus to CPN/FPN, which are located entirely within the CAV1 expression domain. We have now clarified this point, both within the figure legend and the Results text.

- We apologize for this inadvertent exclusion in our statement. We have now modified the figure legend to state that we simultaneously injected CTB-647 into the ipsilateral premotor cortex and CTB-555 into the contralateral somatosensory cortex.

- We have outlined the brains in A-D, which enhances the clarity of the images even better than overlying DAPI.

- Different ages were employed in the retrograde labeling experiments to optimally target each population, based on knowledge of their developmental axonal trajectories, and to replicate previous studies (e.g. Cederquist et al, J Neuro 2013; Sohur et al, Cer Cortex 2014). We now explain our retrograde labeling strategy in more detail within the Methods.

- We have now included schematics of the CTB injection sites for the BPN, CStrPN, and AC. We thank the reviewers for this helpful suggestion.

- We have outlined the brain in panel E', as suggested. For all of our retrograde labeling experiments, our approaches minimized surgical time and insult. As such, morbidity was very low, and our survival rate was near 100%. We have now added this information to the Methods, at the end of the retrograde labeling section.

- We thank the reviewers for the helpful suggestion to add larger representative sections from E”. However, due to the other additions to this already large and complicated figure, we did not find this to be optimally feasible. Further, the example images of CPN/FPN and CAV1 in F serve a similar purpose in demonstrating the distribution of these retrogradely-labeled populations. We respectfully submit that these satisfy the aim of this constructive suggestion.

- We have provided larger high-magnification images of the insets in Panel F, and enlarged the arrows. The N for the quantification analysis was 4 brains (with 4 cortical areas quantified in each brain). We have added this information to the figure legend in addition to the Methods.

Fig.5:

- Panel A: It is not clear where these images are taken from in the cortex. It would also help to provide higher magnification images to show specific cellular components, particularly with respect to the localisation of CAV1 expression.

- Panel B: there is no context of cortical location. The authors describe the cellular location of CAV1 protein in neurons of pups electroporated at E15.5 and collected at P8, but in the manuscript and in the figure legend the authors state that the pups were collected at P6? It would also help to show higher magnification images (as per panel A). This panel is also referred to as Figure 4B in the text.

- Panel C: is referred to as panel D in the manuscript.

- The images in Panel A were taken within S1, and we have now clarified this with a label on the figure, and in the figure legend. We have also added higher magnification images of the soma (A'), in addition to the dendritic tufts (A“).

- We have added lower magnification images to show the cortical location of the electroporations (B and C), and we now indicate the regions shown in the higher magnifications (now B',C'). We have added additional higher magnification images in black and white to more clearly show the cellular localization of CAV1 compared to GFP (B”).

- We have corrected the age of analysis post-electroporation (P6), and the references to the panels within the text.

In Figure 5A, CAV1 is shown to be localized around the soma, apical dendrite and dendritic tuft at P3. Does the expression of CAV1 change over development, for example, what is the subcellular localization of CAV1 at P8? This would suggest possible different roles of CAV1 at different developmental stages.

- We thank the reviewers for highlighting this important consideration. The subcellular expression of CAV1 is quite consistent throughout development. We have clarified this within the text.

Fig.6:

- Table describing t-test results on page 11: Do the bottom two lines refer Fig 6I and not 6H?

- The bottom two lines do refer to Fig 6I rather than 6H. We have corrected this error.
